# Transdermal delivery of gentamicin using dissolving microneedle arrays for potential treatment of neonatal sepsis

**DOI:** 10.1016/j.jconrel.2017.07.032

**Published:** 2017-11-10

**Authors:** Patricia González-Vázquez, Eneko Larrañeta, Maelíosa T.C. McCrudden, Courtney Jarrahian, Annie Rein-Weston, Manjari Quintanar-Solares, Darin Zehrung, Helen McCarthy, Aaron J. Courtenay, Ryan F. Donnelly

**Affiliations:** a Queen's University Belfast, School of Pharmacy, Medical Biology Centre, 97 Lisburn Road, Belfast BT9 7BL, Northern Ireland, United Kingdom; b PATH, PO Box 900922, Seattle, WA 98109, United States

**Keywords:** Neonatal sepsis, Gentamicin, Microneedle

## Abstract

Neonatal infections are a leading cause of childhood mortality in low-resource settings. World Health Organization guidelines for outpatient treatment of possible serious bacterial infection (PSBI) in neonates and young infants when referral for hospital treatment is not feasible include intramuscular gentamicin (GEN) and oral amoxicillin. GEN is supplied as an aqueous solution of gentamicin sulphate in vials or ampoules and requires health care workers to be trained in dose calculation or selection of an appropriate dose based on the patient's weight band and to have access to safe injection supplies and appropriate sharps disposal. A simplified formulation, packaging, and delivery method to treat PSBI in low-resource settings could decrease user error and expand access to lifesaving outpatient antibiotic treatment for infants with severe infection during the neonatal period. We developed dissolving polymeric microneedles (MN) arrays to deliver GEN transdermally. MN arrays were produced from aqueous blends containing 30% (w/w) of GEN and two polymers approved by the US Food and Drug Administration: sodium hyaluronate and poly(vinylpyrrolidone). The arrays (19 × 19 needles and 500 μm height) were mechanically strong and were able to penetrate a skin simulant to a depth of 378 μm. The MN arrays were tested *in vitro* using a Franz Cell setup delivering approximately 4.45 mg of GEN over 6 h. Finally, three different doses (low, medium, and high) of GEN delivered by MN arrays were tested in an animal model. Maximum plasma levels of GEN were dose-dependent and ranged between 2 and 5 μg/mL. The time required to reach these levels post-MN array application ranged between 1 and 6 h. This work demonstrated the potential of dissolving MN arrays to deliver GEN transdermally at therapeutic levels *in vivo*.

## Graphical abstract

**Figure:**
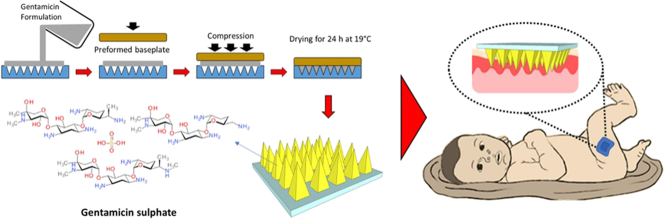

## 1 Introduction

Neonatal infections, including sepsis, are a significant cause of childhood mortality in low-resource settings; they cause an estimated 26% to 36% of the neonatal deaths that occur globally in low- to middle-income countries [1], [2]. Approximately 421,000 newborns die each year from sepsis, primarily in low-resource settings [3]. Recently updated WHO guidelines for outpatient treatment of severe bacterial infections (sepsis) in neonates (0–28 days old) and young infants (0–59 days old) when referral for hospital treatment is not feasible include intramuscular (IM) gentamicin and oral amoxicillin [4].

GEN is a polar, water-soluble compound, which presents very poor dermal and intestinal permeability. GEN for injection is presented as an aqueous solution of gentamicin sulphate in vials or ampoules. It has a narrow therapeutic index and, like other aminoglycosides, it is potentially ototoxic and nephrotoxic. GEN is excreted almost entirely unchanged by the kidney, mostly by glomerular filtration and has a short plasma elimination half-life (2 − 3 h) in adults with normal renal function [5]. The elimination half-life of GEN in neonates and children depends on the patient age [6], [7], [8]. Consequently, when this drug is administered to neonates, careful dose calculation and close monitoring are required to prevent GEN-induced toxicity. Ideally, close monitoring of GEN concentration in serum should be conducted, but this is not commonly available in low-resource settings and is currently not part of the WHO recommendations specifically for the outpatient setting.

An easy-to-use, less-invasive, affordable delivery method for GEN paired with oral amoxicillin has the potential to expand access to lifesaving outpatient antibiotic treatment for infants with severe infection during the neonatal period. Dissolving MNs are drug delivery systems with potential to administer GEN while fulfilling these requirements [9], [10], [11]. They are made of safe water-soluble biodegradable or biocompatible polymers that act as a matrix containing the drug to be delivered. Following insertion into skin and subsequent contact with skin interstitial fluid, MNs dissolve and release the encapsulated drug. This MN system has successfully delivered numerous substances, including low molecular weight (MW) drugs [12], [13], macromolecules [14], [15], [16], [17], DNA [18], and vaccines [19], [20], [21]. Recently, the work of our research group has focused on delivering drugs at clinically-relevant doses using MN arrays [22], [23]. McCrudden et al. have formulated and tested dissolving MNs for delivery of therapeutically-relevant concentrations of ibuprofen sodium [24]. In addition, *in vitro* delivery of a combination of cardiovascular drugs from a single-dissolving MN array has recently been investigated by Quinn et al. [25].

GEN delivery by a dissolving MN array could enable administration in both inpatient and outpatient settings. Compared to parenteral delivery, this novel delivery system could reduce training requirements for calculation of GEN dose and administration, thereby expanding access to antibiotics to treat neonates with possible serious bacterial infections, particularly in resource-limited settings. The present work evaluates the possibility of using dissolving MN arrays for the delivery of GEN. To the best of our knowledge, this is the first study that uses dissolving MN arrays for the delivery of therapeutically relevant doses of an antibiotic.

## 2 Material and methods

### 2.1 Materials

*N*-acetylcysteine (NAC), methanol (HPLC grade), poly(vinyl alcohol) (PVA) (80% hydrolysed, MW = 9000–10,000 Da), poly(vinylpyrrolidone) (PVP), (MW 360 kDa) and poly(ethylene glycol) (PEG) (MW 400 Da) were obtained from Sigma-Aldrich (Dorset, UK). Gantrez® S-97 (PMVE/MA) (a copolymer of methyl vinyl ether and maleic acid, MW 1,500,000 Da) and plasdone® K-29/32 (PVP) (MW 58 kDa) were kindly donated by Ashland (Kidderminster, UK). Glycerol bidistilled (AnalaR NORMAPUR®, 99.5%) and sodium chloride (AnalaR NORMAPUR® ACS) were obtained from VWR International (Leicestershire, UK). Gentamicin sulphate and *o*-phthaldialdehyde (OPA) were obtained from Tokyo Chemical Industry UK Ltd. (Oxford, UK). Heparin Sodium (200 units in 2 mL) was provided by Wockhardt® UK Ltd. (Wrexham, UK). Hyabest®(S) LF-P (sodium hyaluronate 99.9% purity, MW 250–400 kDa range) was obtained from Kewpie Corporation Fine Chemical Division (Tokyo, Japan).

### 2.2 Methods

#### 2.2.1 Fabrication of dissolving MNs

**Fig. 1:**
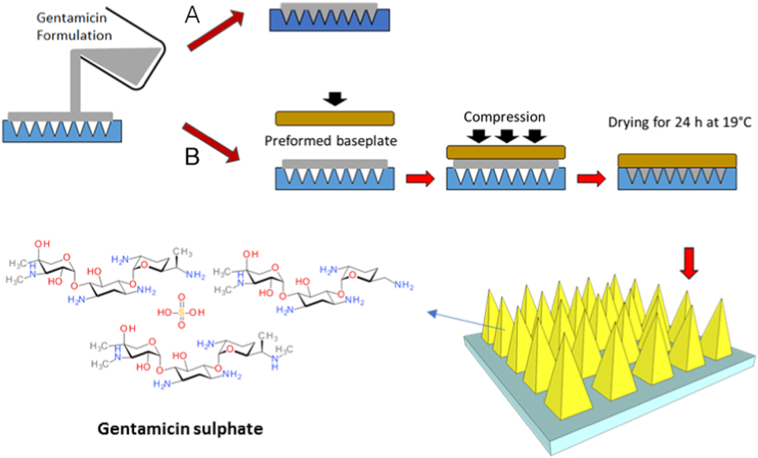
Diagrammatic representation of MN fabrication process in (A) one-step and (B) two-step.

Selected polymers were mixed with GEN and dissolved in deionized water as potential matrices in the formation of MN. The blend was then mixed and sonicated at 37 °C for 1–2 h depending on the composition. The resulting formulations (100 mg) were poured into MN moulds and positive pressure (3–4 bar) was applied for 15 min to fill the moulds. MN arrays were allowed to dry for 24 h in a controlled temperature room at 19 °C. The MN array design used to cast the formulations was an array consisting of 361 pyramidal needles (19 rows of 19 needles) each with heights of 500 μm and array area of 0.45 cm^2^. In addition, a two-step method was investigated with different dissolving MN formulations. For this purpose, aqueous blends (100 mg) containing GEN were poured into MN moulds. Post-casting, a preformed baseplate, prepared as detailed in Section 2.2.2, was immediately added on top of the GEN formulation. Positive pressure (3–4 bar) was applied for 15 min to fill the moulds and then MNs were dried for 24 h at 19 °C. Once dried, dissolving MNs were visually inspected using a Leica EZ4D digital light microscope (Leica Microsystems, Milton Keynes, UK). Fig. 1 shows a diagrammatic representation of the fabrication method.

#### 2.2.2 Preparation of films as a backing layer for dissolving MNs

Thirty grams of formulation containing 15% w/w PVP (MW 360 kDa) were poured into 10 × 10 cm moulds consisting of a release liner with the siliconised surface facing upwards secured to a Perspex® base with wing nuts. Previously, formulation was centrifuged at 3500 rpm (rpm) for 15 min to ensure removal of entrapped air. Films were placed on a levelled surface and required at least 48 h to dry at room temperature. Once dried, the film was cut into small portions of approximately 1 cm^2^.

#### 2.2.3 Microneedle mechanical characterisation

The mechanical strength of dissolving MN arrays was investigated by using a TA.XT2 Texture Analyser (Stable Micro Systems, Haslemere, UK) in compression mode, as previously described [26]. Briefly, MN arrays were attached to the moveable cylindrical probe (length 5 cm, cross-sectional area 1.5 cm^2^) of the Texture Analyser and the probe was programmed to move vertically downward at a rate of 1.19 mm/s. The test station compressed MN arrays against a flat block of aluminium of dimensions 9.2 × 5.2 cm, at defined rates for 30 s using a force of 32 N/array [27]. The Leica EZ4D digital microscope was utilised for visualisation of MN arrays before and after application of the compression load. The heights of individual MNs were measured before and after testing, using the ruler function of ImageJ® software (US National Institutes of Health, Bethesda, Maryland, USA) so that the percentage change in MN height could be calculated using Eq. (1), where, H_BC_ is the Height before compression and H_AC_ is the Height after compression.

(1) $$\%\text{Compression}=\frac{H_{\mathrm{BC}}-H_{\mathrm{AC}}}{H_{\mathrm{BC}}}\;x\;100$$

In order to evaluate MN insertion, the Parafilm M® (Bemis Company Inc., Soignies, Belgium) insertion model developed by Larrañeta et al. was used [27]. MN arrays were inserted using the Texture Analyser, at 32 N/array for 30 s, into eight-layer folded Parafilm M® sheets. After the insertion, the MN arrays were removed from the polymeric layers. Parafilm M® layers were unfolded and the number of holes in each layer was evaluated using the digital microscope and two polarizer filters.

#### 2.2.4 Dissolution studies and recovery of GEN content from dissolving MNs

Dissolution kinetics and drug content evaluations were carried out on candidate GEN-loaded dissolving MN arrays to ensure that there were no compatibility issues between GEN and the polymers selected as a matrix for MN formation. Individual MNs were placed in a 20 mL volume of phosphate buffered saline (PBS), pH 7.4 in a glass vial. The time required for complete dissolution of the sample using continuous stirring at 600 rpm at 37 °C was recorded. Following appropriate dilutions, the GEN content obtained from the dissolving MNs was determined by reversed-phase high-performance liquid chromatography (RP-HPLC), as detailed in Section 2.2.8.

#### 2.2.5 In-skin dissolution kinetics of dissolving MNs

Optical coherence tomography (OCT) (EX1301 OCT microscope, Michelson Diagnostics Ltd., Kent, UK) was employed to investigate the dissolution rate of dissolving MNs in skin, as previously described [28]. Neonatal porcine skin was used as a skin model, due to its similarities to human skin in terms of general structure, thickness, hair density, pigmentation, collagen and lipid composition [29]. Neonatal porcine skin samples were obtained from stillborn piglets and immediately (< 24 h after birth) excised and trimmed to a thickness of 500 μm using an electric dermatome instrument (Integra® Life-Sciences Corporation, Ratingen, Germany). Skin samples were stored in sealed Petri dishes at − 20 °C until use. Skin samples were shaved and equilibrated in PBS, pH 7.4 for 15 min prior to use. One section of 500 μm thick neonatal porcine skin was placed, dermal side facing downwards, onto a 500 μm piece of Suprasorb® G wound dressing (Lohmann & Rauscher GmbH, Rengsdorf, Germany). The Suprasorb® G wound dressing was equilibrated for 48 h in a copious volume of PBS, pH 7.4 at 37 °C prior to use. The piece of Suprasorb® G wound dressing was pinned, using drawing pins, into a Styrofoam™ platform which was wrapped in aluminium foil and Parafilm M®. The skin was pinned on top of these wound dressing sections. Dissolving MN arrays were adhered to a piece of Sellotape® and manually applied into the skin. This experimental design maintained a hydrated membrane. The swept-source Fourier domain OCT system has a laser centre wavelength of 1305.0 ± 15.0 nm, facilitating real-time high-resolution imaging of the upper skin layers (7.5 μm lateral and 10.0 μm vertical resolution). The skin was scanned at a frame rate of up to 15 B-scans (2D cross-sectional scans) per second (scan width = 2.0 mm). The 2D images were examined using the imaging software ImageJ®. To allow differentiation between MN and skin layer, false colours were applied to images using Microsoft® PowerPoint 2016 (Microsoft Corporation, Redmond, Washington, USA). OCT images were captured at designated time points over the course of a 1 h experiment.

#### 2.2.6 *In vitro* permeation study of GEN from dissolving MNs

Transdermal permeation of GEN released from dissolving MNs was investigated *in vitro* using Franz diffusion cells, as described previously [14], [24]. A section of the neonatal porcine skin (350 μm thick), obtained as described above, was secured to the donor compartment of the diffusion cell using cyanoacrylate glue with the *stratum corneum* facing upwards in the donor compartment. MNs were inserted into the centre of the skin section by applying manual pressure for 30 s to a syringe plunger, with the flat end pressing on the baseplate. A stainless-steel cylinder (diameter 11.0 mm, 5.0 g mass) was then placed on top of the MNs to hold them in place. The donor compartments were set onto the receptor compartments of the Franz cells, which were sealed using Parafilm M® to reduce evaporation. In the receiver solution, PBS (pH 7.4) was thermostatically maintained at 37 ± 1 °C and stirred at 600 rpm. Samples (≤ 200 μL) were removed from the sampling arms of the Franz cells at predetermined time intervals (15 min, 30 min, 1 h, 2 h, 3 h, 4 h, 5 h, 6 h and 24 h) using 1 mL syringes with 8 cm needles and 200 μL of PBS was subsequently added to replace the volume taken. Derivatisation of the samples was carried out using volumes of 100 μL of sample: 100 μL of derivatising reagent. Samples were centrifuged for 10 min and then filtered through 0.2 μL syringe filters. Finally, the samples were analysed by RP-HPLC.

#### 2.2.7 *In vivo* delivery of GEN

**Fig. 2:**
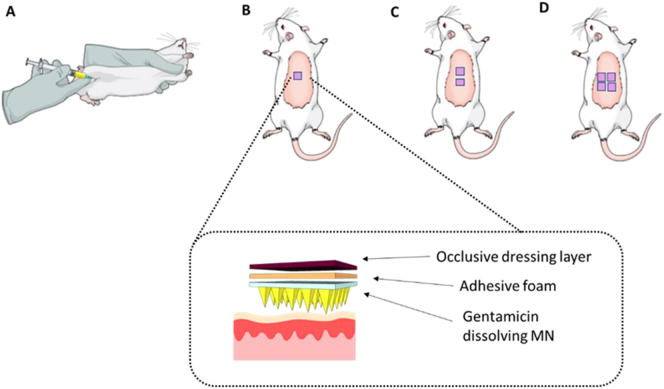
Schematic representation showing the four treatment groups investigated in the *in vivo* experiment. (A) IM administration of GEN, 7.5 mg/kg. Transdermal application of (B) one, (C) two and (D) four MN arrays to the hairless back of the animals and the setup used to ensure the MNs were kept in place.

An *in vivo* experiment using dissolving MNs was carried out to evaluate profiles of GEN in plasma after the application of MN arrays. Female Sprague-Dawley rats weighing 208.65 ± 21.48 g and aged 10 weeks were acclimatised to laboratory conditions for a 7-day period. Animals were separated in four groups (*n* = 10 per group) (Fig. 2). In the control group (A), animals received an IM injection of GEN based on individual rat weight, 7.5 mg/kg of gentamicin sulphate to mimic the WHO higher neonatal dose [4]. Thus, a solution of 15 mg/mL of GEN was freshly prepared in sterile water for injection. The IM injection was given into the muscles of the thigh and the volume injected was ≤ 100 μL. The transdermal treatments groups were denominated: low dose (B), medium dose (C) and high dose (D), in which rats were treated with one, two or four dissolving MNs, respectively. Dissolving MN arrays were prepared to contain an average of 30 mg of GEN per array.

Animals were anaesthetized using gas anaesthesia (2–4% isoflurane in oxygen) and the backs of the rats were shaved using an animal hair clipper and hair removal cream, prior to application of MNs. Dissolving MNs were secured onto an open “frame” of adhesive foam and were applied using firm finger pressure onto a pinched section of skin on the back of the animals. To keep the MN array in place, an occlusive dressing layer, Tegaderm™ (3 M, St Paul, Minnesota, USA), was placed on top of the dissolving MNs, and Micropore™ tape (3 M UK Plc, Bracknell, Berkshire, UK) was used to wrap the back of the animals. Blood samples (≤ 200 μL) were collected into heparinised tubes at designated time points: 1, 2, 4, 6 and 24 h *via* tail vein bleeds. The MN arrays were kept in place for 24 h. It was not possible to track plasma level of GEN at each time point from the same animal due to restrictions in blood sampling volumes by our Institutional Project Licence. To overcome this, blood samples were taken from the first five rats at 1 h and 4 h. The other five animals were bled at 2 h and 6 h and all animals (*n* = 10) were sampled at 24 h. After collection of rat blood, plasma separation was performed by centrifuging the blood at 3000 relative centrifugal force (RCF) for 10 min at 4 °C in a refrigerated centrifuge (Z 216 MK, HERMLE Labortechnik GmbH, Wehingen, Germany). Plasma samples were collected in 0.5 mL microtubes and stored in a freezer at − 80 °C prior to analysis. To extract GEN, plasma samples were vortexed for 10 s and transferred to Amicon® Ultra-0.5 centrifugal filter devices (10 kDa) (EMD Millipore Corporation, Billerica, Massachusetts, USA). Samples were centrifuged at 15,000 RCF for 30 min at 4 °C to remove proteins prior to HPLC analysis. A pre-column derivatisation was carried out with the filtered samples obtained after centrifugation. In all cases, the ratio of derivatising reagent mixture and samples was 1:1, depending on the volume of sample recovered. Approval for animal experiments was obtained from the Committee of the Biological Research Unit, Queen's University Belfast. The work was carried out under Project Licence PPL 2794 and Personal Licence PIL 1466. All *in vivo* experiments were conducted according to the policy of the federation of European Laboratory Animal Science Associations and the European Convention for the protection of vertebrate animals used for experimental and other scientific purposes, with implementation of the principles of the 3Rs (replacement, reduction, and refinement). Values for area under the curve (AUC) were calculated by the trapezoidal method using GraphPad Prism®.

#### 2.2.8 GEN analytical method

Chemical derivatisation of GEN components was accomplished to increase sensitivity and allow low level quantification by RP-HPLC. The derivatisation of GEN was carried out using *o*-phthaldialdehyde (OPA) and *N*-acetylcysteine (NAC) as reagents by slight modification of the procedure developed by Kowalczuk et al. [30]. Briefly, the derivatisation reagents were prepared by dissolving 20 mg of OPA in 1 mL of methanol and 100 mg of NAC was dissolved in 1 mL of deionized water. To the mixture of OPA and NAC, 8 mL of 0.05 M borate buffer (pH 9.3) were added. OPA and NAC were freshly prepared for each derivatisation. For GEN quantification, 500 μL of sample was reacted with 500 μL of derivatising reagent mixture in microtubes in a water-bath at 50 °C for 20 min. After cooling, samples were analysed by RP-HPLC. The mobile phase was a mixture of methanol and an aqueous solution of 0.02 M of sodium hexanesulphonate and glacial acetic acid (65.5:33.5, v/v), with a flow rate of 1 mL/min, an injection volume of 40 μL and a run time of 25 min per sample. GEN was detected using fluorescence detection with excitation wavelength set at 328 nm and emission at 423 nm. The gain was optimal at a reading of 12. Phase separation was performed on a Phenomenex® SphereClone™ 5 μm ODS (1) column (150 mm × 4.60 mm with 5 μm packing; Phenomenex, Cheshire, UK) at ambient temperature. All analytical runs were preceded by a security guard cartridge of matching chemistry. Data acquisition and analysis were performed using Agilent Rapid Res® software (Agilent Technologies UK Ltd., Stockport, UK). All samples were centrifuged at 14,000 rpm using an Eppendorf MiniSpin® centrifuge (Eppendorf UK Limited, Stevenage, UK) for 10 min prior to HPLC analysis. Unknown concentrations of test samples were calculated using external standards.

The HPLC method for quantification of GEN substances in PBS and rat plasma were developed and validated in accordance with the International Committee of Harmonisation Q2(R1) guidelines for validation of analytical procedures [31]. Table 1 shows the slope, y-intercept and coefficient of determination (R^2^ value) obtained from least squares linear regression analysis, followed by correlation analysis of the calibration plots for derivatised GEN in PBS and plasma.

**Table 1:** Calibration curve properties for derivatised GEN quantification in PBS, pH 7.4 and rat plasma and limits of detection (*LoD*) and quantification (*LoQ*) for GEN.

	Slope	y-Intercept	R^2^	*LoD* (μg/mL)	*LoQ* (μg/mL)
PBS, pH 7.4	291.46	− 2.366	0.9987	0.049	0.149
Rat plasma	670.98	− 92.982	0.9955	0.099	0.301

#### 2.2.9 Statistical analysis

All data were expressed as means ± standard deviation. The statistical analyses were performed using GraphPad Prism® version 5.3 (GraphPad Software, San Diego, California, USA). Where appropriate, the Mann-Whitney *U* test was performed for comparison of two groups, when *n* < 5. The unpaired *t*-test was used for comparison of two groups, when *n* > 5 and data were normally distributed. The Kruskal-Wallis test with *post-hoc* Dunn's test was used for comparison of multiple groups. In all cases, *p* < 0.05 was used to denote statistical significance.

## 3 Results

### 3.1 Formulations investigated in the production of GEN-loaded dissolving MNs

Several polymers were investigated to evaluate their potential as matrices in the formation of dissolving MNs loaded with a high concentration of GEN. Some of the selected polymers, such as PVP high MW (360 kDa) or PVA were not able to form a homogeneous aqueous blend with a high concentration of GEN. In contrast, sodium hyaluronate (HA), MW 250–400 kDa range, in combination with low MW PVP (58 kDa) formed the most promising formulations. Low concentrations of glycerol were used in some formulations as plasticisers. Dissolving MNs that appeared to be fully formed upon visual examination under a light microscope (Table 2) were chosen for further dissolution and drug content evaluations.

**Table 2:** Formulations selected for the production of GEN loaded dissolving MNs.

Formulation	Composition (% w/w)				MN morphology
	GEN	HA	PVP	Glycerol	
F1	30	3.4	1	–	
F2	30	3	1	0.5	
F3	30	3	2	0.5	
F4	30	5	5	–	
F5	30	5	2.5	–	
F6	30	3.4	1	0.5	

### 3.2 Mechanical characterisation of dissolving MN arrays

**Fig. 3:**
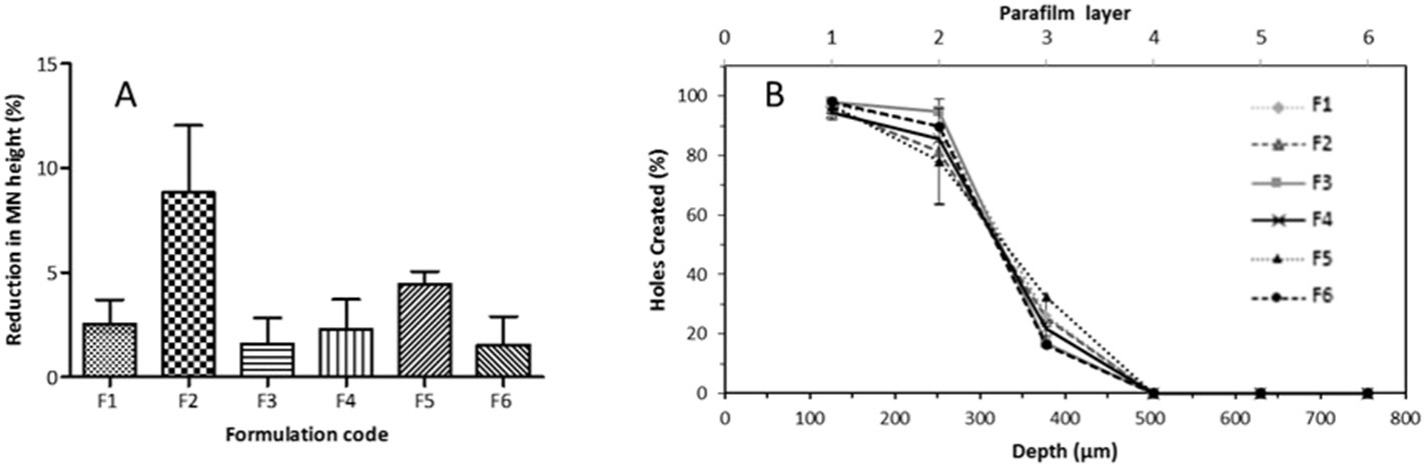
(A) Comparison of percentage of MN height reduction of dissolving MN formulations tested, observed following the application of a force of 32 N/array (means + S.D., *n* = 4). (B) Percentage of holes created in each Parafilm M® layer following insertion of dissolving MN formulations investigated. MN arrays used were 19 × 19 with a needle height of 500 μm, base width of 300 μm and a base interspacing of 50 μm (means + S.D., *n* = 4).

Dissolving MNs prepared with the selected formulations were used to investigate the effects of compression tests on the heights of individual needles on the MN arrays. Fig. 3A shows the reduction in MN height for all the selected formulations. The application force of 32 N/array reduced the height of all dissolving MNs. The percent reduction in MN height for formulation F2 and F5 was < 9% and < 5%, respectively. The percent reduction in the original MN height was < 2% for formulations F3 and F6 and < 3% for formulations F1 and F4. The percent reduction of F2 was significantly different compared to all other formulations (*p* = 0.0084).

The percentage of holes created in each Parafilm M® layer for different dissolving MN formulations is shown in Fig. 3B. The *in vitro* insertion depths obtained for formulations tested showed a similar trend and all were capable of piercing three layers of Parafilm M®. The average thickness of a Parafilm M® layer is 126 ± 7 μm; this suggests that MNs were inserted up to 378 μm of the total 500 μm height, approximately 75.6% of MN tip height. These results indicate that total needle length inserted correlated with previously reported studies, where the insertion was equivalent to 60% of the total needle length [27], [32]. However, it is important to note that formulations F2, F3, F4 and F5 were not selected for further investigation because these formulations showed some individual needles remained in the Parafilm M® after the insertion test.

### 3.3 Fabrication of MN in two-steps

**Fig. 4:**
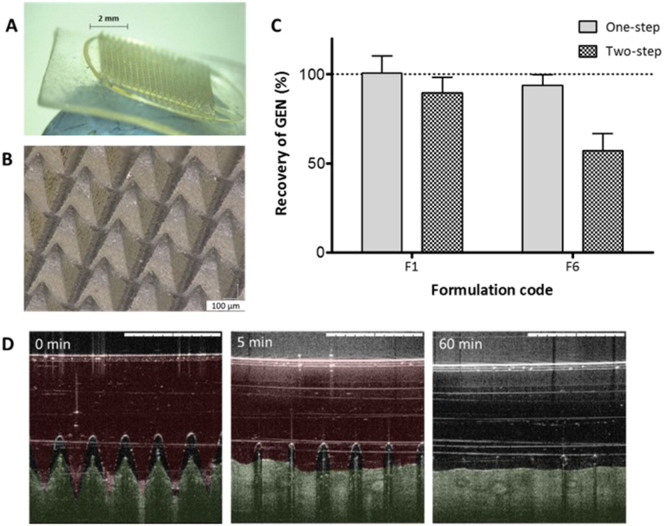
Digital pictures showing two-layered MN array taken with (A) the Leica EZ4D digital microscope and (B) the Keyence VHX-700F digital microscope (Keyence, Milton Keynes, UK). (C) Comparison of percentage recovery of GEN from dissolving MNs prepared from F1 and F6 in one-step and two-step (means ± S.D., *n* = 3). (D) Representative OCT images showing *in vitro* dissolution kinetics of a 19 × 19 MN array prepared using F1 and inserted manually in neonatal porcine skin. False colours were applied to the skin and MNs. The original OCT images can be found on the Supplementary content (Fig. S1). The white scale bar at top right represents a length of 1 mm. Digital pictures showing two-layered MN array taken with (A) the Leica EZ4D digital microscope and (B) the Keyence VHX-700F digital microscope (Keyence, Milton Keynes, UK). (C) Comparison of percentage recovery of GEN from dissolving MNs prepared from F1 and F6 in one-step and two-step (means ± S.D., *n* = 3). (D) Representative OCT images showing *in vitro* dissolution kinetics of a 19 × 19 MN array prepared using F1 and inserted manually in neonatal porcine skin. False colours were applied to the skin and MNs. The original OCT images can be found on the Supplementary content (Fig. S1). The white scale bar at top right represents a length of 1 mm.

After optimising the formulation of the MN, a two-step manufacturing process was tested to provide a degree of flexibility to the baseplate of the MN array. Fig. 4A and B shows a two-layered MN array.

Formulation F6, which contained 0.5% glycerol to reduce fragility, and prepared with a baseplate made of 15% w/w of PVP, MW 360 kDa: 2.5% glycerol, showed a remarkable reduction of recovery of drug compared to F1 fabricated in two-step which contained no glycerol in the needles or baseplate (Fig. 4C). The percentage of GEN recovery was found to be 89.56 ± 8.82% and 57.27 ± 9.38% for the two-layered MNs prepared using F1 and F6, respectively. However, the percentage of GEN recovery was 100.7 ± 9.69% and 89.56 ± 8.82% for formulations F1 and F6 prepared as a single layer. Besides, MNs containing glycerol in the preformed baseplate became flexible, losing mechanical strength, after 10 days of storage at room temperature. This was unsurprising given the highly hygroscopic nature of glycerol. Consequently, F6 was discarded. MN arrays prepared in two-step, made of F1 in the needles and 15% w/w of PVP high MW (360 kDa) as a preformed baseplate showed adequate recovery of GEN and desirable mechanical strength. Fig. 4D displays the *in vitro* dissolution kinetics of F1 in neonatal porcine skin using OCT. MN arrays prepared from F1 effectively pierced the skin and the in-skin MNs dissolved in the first five min. After 1 h, skin was completely recovered. Consequently, this formulation was taken forward for *in vitro* permeation studies to investigate the efficacy of drug delivery. Formulation F1 retained its mechanical strength properties after storage in a plastic container covered with aluminium foil at 19 °C for > 4 weeks.

### 3.4 *In vitro* permeation study of GEN from dissolving MNs

**Fig. 5:**
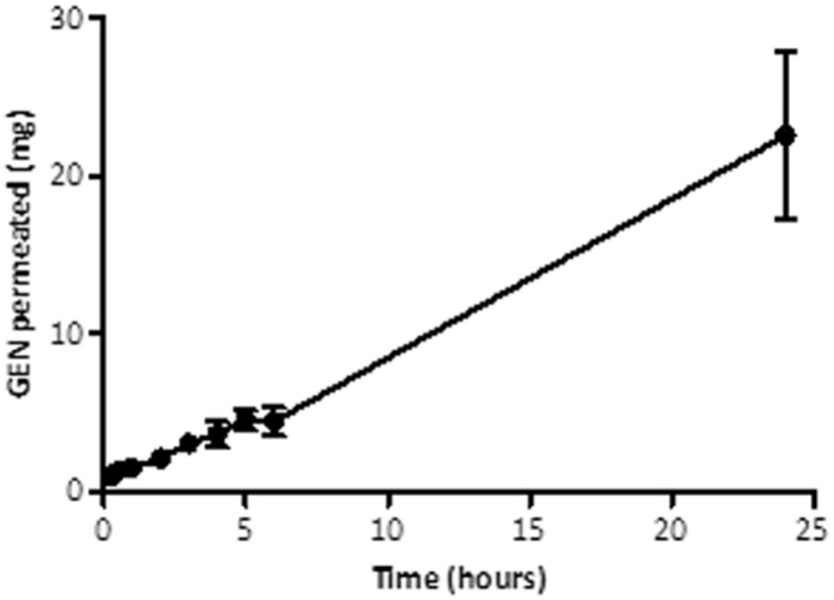
*In vitro* permeation profile of GEN across 350 μm neonatal porcine skin when delivered from dissolving MNs made of formulation F1 over a 24 h period. Means ± S.D., *n* = 4.

Two-layered MNs were fabricated from formulation F1 containing approximately 30 mg of GEN per array. The amount of GEN loaded in the needles was approximately 4.8 mg, calculated considering the density of the formulation and the volume of the 361 square pyramidal needles. These arrays were used to evaluate the *in vitro* permeation of GEN across neonatal porcine skin (Fig. 5). Dissolving MNs made of F1 delivered 1.53 ± 0.28 mg of GEN in 1 h, 3.08 ± 0.5 mg of GEN in 3 h (10.25%) and a total of 22.56 ± 5.36 mg of GEN in 24 h (75%), delivered across the neonatal porcine skin. All GEN loaded in the needles was delivered within 6 h and most of the GEN content forming the baseplate was delivered within 24 h.

### 3.5 *In vivo* delivery of GEN

**Fig. 6:**
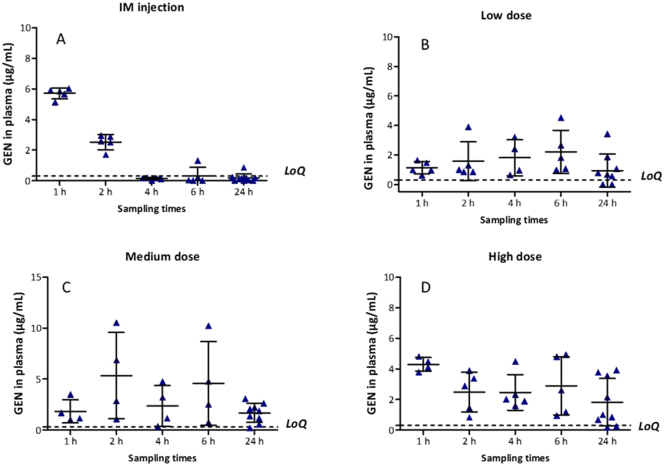
(A) *In vivo* plasma profile of GEN following IM injection of gentamicin sulphate dissolved in sterilised water for injections (dose was 7.5 mg/kg). Means ± S.D., *n* = 5 at 1 h, 2 h, 4 h and 6 h; *n* = 10 at 24 h. (B) Transdermal delivery by using one dissolving MN (low dose). Means ± S.D., *n* = 5 at 1 h, 2 h and 6 h; *n* = 4 at 4 h; *n* = 10 at 24 h. (C) Transdermal delivery by using two dissolving MNs (medium dose). Means ± S.D., *n* = 4 at 1 h, 2 h, 4 h and 6 h; *n* = 9 at 24 h. (D) Transdermal delivery by using four dissolving MNs (high dose). Means ± S.D., *n* = 4 at 1 h; *n* = 5 at 2 h, 4 h and 6 h; *n* = 10 at 24 h. Dashed lines represent limit of quantification for GEN (*LoQ* = 0.30 μg/mL).

The *in vivo* plasma profile of GEN post-IM injection is shown in Fig. 6A. Therapeutic levels of GEN in plasma (5.72 ± 0.35 μg/mL) were achieved within an hour following IM injection. The mean plasma level of GEN progressively decreased to 2.52 ± 0.49 μg/mL at 2 h, after which most concentrations were found to be below the *LoQ*.

The mean concentration of GEN in plasma for the low dose treatment group was 1.13 ± 0.42 μg/mL at 1 h, 1.58 ± 1.31 μg/mL at 2 h and 1.80 ± 1.22 μg/mL at 4 h. A maximal concentration of GEN (2.21 ± 1.46 μg/mL) was achieved at 6 h and then was slightly reduced (0.93 ± 1.11 μg/mL) at 24 h (Fig. 6B).

The mean concentration of GEN in plasma for the medium dose treatment group increased from 1.83 ± 1.13 μg/mL at 1 h to 5.34 ± 4.23 μg/mL at 2 h. The mean GEN concentration in plasma dropped to 2.37 ± 1.81 μg/mL at 4 h, increased again to 4.58 ± 4.11 μg/mL at 6 h and dropped to 1.68 ± 0.94 μg/mL at 24 h (Fig. 6C).

The plasma profile for the high dose treatment group is presented in Fig. 6D. A therapeutic concentration of GEN in plasma was achieved within an hour (4.30 ± 1.47 μg/mL). After 1 h, the mean drug concentration in plasma dropped and remained consistently < 3 μg/mL for subsequent sampling points.

**Fig. 7:**
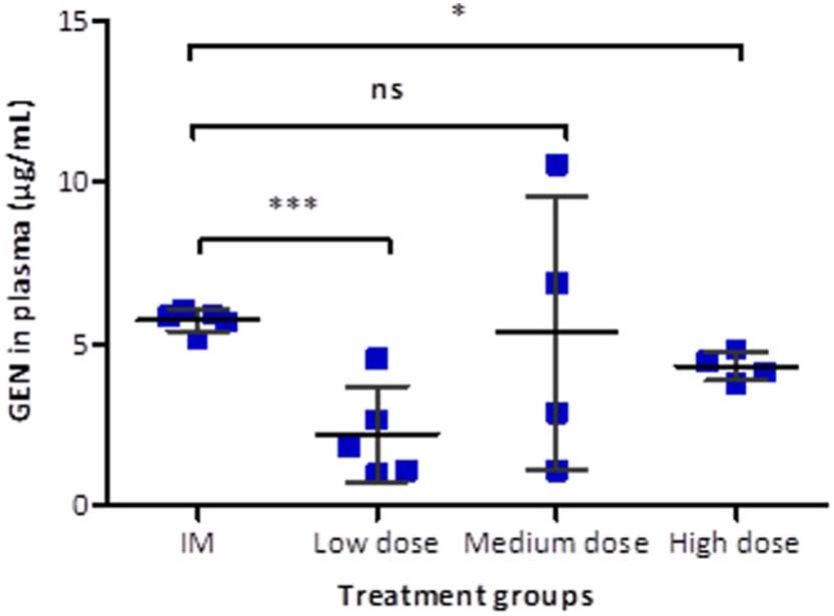
Comparison of mean peak plasma concentrations of GEN (Cmax) following IM injection and transdermal delivery of GEN at increasing doses. Means ± S.D., *n* = 5 for the control group and the low dose and *n* = 4 for medium and high dose. (ns) indicates *p* values > 0.05, (*) indicates *p* values ≤ 0.05 and (***) indicates *p* values ≤ 0.001.

The IM control treatment group presented the highest plasma concentration of GEN at 1 h (Cmax = 5.72 ± 0.35 μg/mL) (Fig. 7). The Cmax for the IM control group was statistically higher than those for the low dose Cmax (*p* = 0.0008) and the high dose treatment group Cmax (*p* = 0.0159). However, the maximum plasma concentration of GEN for the medium dose transdermal treatment group was not different compared to the IM control Cmax (*p* = 1.000). Pharmacokinetic parameters are presented in Table 3.

**Table 3:** Pharmacokinetic parameter of GEN applied to rats for the IM injection and the three transdermal treatments groups: low dose, medium dose and high dose. Means ± S.D., *n* = 5 for the IM group and low dose and *n* = 4 for medium and high doses.

Parameter	IM injection	Low dose	Medium dose	High dose
AUC (μg. h/mL)	12.03	37.05	74.58	56.13
Tmax (h)	1	6	2	1
Cmax (μg/mL)	5.72 ± 0.35	2.21 ± 1.46	5.34 ± 4.23	4.30 ± 1.47

As expected, the lowest GEN Cmax was observed following administration of an individual dissolving MN patch and the time of maximum plasma concentration (Tmax) was 6 h following application. The comparison between Cmax of the transdermal groups was not statistically significant (*p* = 0.1629). However, increasing the number of GEN MN arrays resulted in an earlier Tmax.

## 4 Discussion

Despite the limitation of drug loading in dissolving MNs, several studies have highlighted the ability of this platform to enhance transdermal delivery of an extensive range of drug molecules [9], [10]. The initial objective of this work was to formulate well-formed MNs using biocompatible materials approved by the US Food and Drug Administration (FDA) with high drug loading of GEN. Ideal GEN MN formulations should have sufficient mechanical strength for a successful application into the skin by the patient or a healthcare provider, rapidly dissolve, and yield high drug recovery. The geometry of the MN and physicochemical properties of the drug and polymers affect mechanical strength [10].

We formulated numerous dissolving MNs using water-soluble biocompatible or biodegradable polymers to investigate their compatibility with a high dose of GEN (30% w/w). Some polymers, such as PVP [25], PVA [33], [34], sodium hyaluronate [35], [36] and Gantrez® copolymers [12], [24], [25], [37], [38] have frequently been used as matrices in the formation of dissolving MNs. PVP of high MW (360 kDa) and PVA (MW 9–10 kDa), showed incompatibilities with high doses of GEN. In contrast, MNs formulated using low concentrations of sodium hyaluronate (MW 300 kDa) and PVP (MW 58 kDa) formed MNs that, upon visual inspection, were fully formed. Plasticisers, such as glycerol and PEG 400, were used to decrease MN brittleness, a property that can prevent successful insertion in skin and thus inhibit drug delivery. Glycerol is commonly incorporated into films to overcome brittleness [39], [40]. Moga et al. have reported the use of plasticisers such as castor oil, Tween® 80, glycerol, PEG 400 and trimethyl citrate to impart flexibility to the blends of a PVP/poly(vinylacetate) copolymer used as a substrate for dissolving MNs [41]. Among all the prepared formulations, a few were selected based on the formation of intact and reproducible MNs possessing rapid dissolution rates (Table 2). The main compounds used to formulate these arrays were PVP, HA and glycerol.

This study was conceived as a proof of concept and the main objective was to explore the possibility of administering therapeutically relevant doses of GEN using dissolving MNs. To the best of our knowledge there are no previous articles describing similar drug delivery systems. Accordingly, we maximise the amounts of GEN loaded in the MN arrays. The arrays were manufactured containing GEN in the needle tips and in a small baseplate. In this way GEN can be administered from the needle tips and subsequently from the baseplate. The drug contained in the baseplate can permeate through the created pores as suggested by McCrudden et al. [24].

The delivery of the drug *via* dissolving MNs depends on their ability to pierce the *stratum corneum*; dissolving MNs must therefore possess sufficient mechanical strength. Accordingly, a range of mechanical characterisation tests has been developed to test their mechanical strength and insertion characteristics. Such tests include MN insertion into skin [42], MN compression test [26] and OCT [28], among others. Lutton and co-workers have recently highlighted an urgent need for consistency to characterise MN products in order to demonstrate MN strength and insertion [43]. The *in vitro* insertion test developed by Larrañeta et al. was used to assess the ability of the dissolving MNs to pierce a skin simulant, Parafilm M®, thus avoiding dissolution of the MNs in biological tissue. The mechanical testing of dissolving MN provided information about which concentrations of HA and PVP yielded the best strength and insertion capabilities. In summary, formulations investigated showed to form robust needles that were not fractured upon compression. However, some formulations were deemed unsuitable for further investigation because needles tips were not entirely formed and also some individual needles retained in the Parafilm M® post-insertion test. Furthermore, dissolving MNs were prepared in two-step to provide certain flexibility to the baseplate of the MN arrays. Ideally, the baseplate should have some inherent flexibility to allow conformation to the skin surface without cracking; a variety of techniques have been used to fabricate dissolving MNs in a two-step process to localise the active compounds to the needles, thereby reducing drug waste [15], [17], [18], [44], [45].

The rate of dissolution of dissolving MNs in-skin depends on the solubility in water of the polymers and drug studied and also the composition [10]. The non-invasive technique based on light reflection, OCT, was used to investigate the in-skin dissolution kinetics of dissolving MNs. This technique has been widely used in clinical care, predominantly for ophthalmic applications, and in research, providing high-resolution cross-sectional images of the object of study [46]. A number of studies have highlighted the potential of OCT as a valuable tool to assess MN insertion in skin, allowing measurement of the MN depth and visualisation of microchannels created in skin [26], [28], [47], [48]. Likewise, OCT has been used for *in vivo* visualisation of dissolution of MNs into murine skin [16], [17] and *in situ* dissolution of MNs into neonatal porcine skin [28]. The *in vivo* swelling of hydrogel-forming MNs has also been examined using OCT in the skin of human volunteers [49], [50], [51] and *in vitro* in neonatal porcine skin [52]. The GEN-loaded dissolving MNs were robust enough to penetrate skin *in vitro* and the complete dissolution of the in-skin MNs was achieved in the first five min.

The *in vitro* permeation studies carried out with two-layered dissolving MNs (F1) across neonatal porcine skin *via* the Franz diffusion cell technique deduced that 14.85% (4.45 mg) of GEN loadings were delivered over a 6 h experimental period and approximately 75% of the GEN contained in the MN array was successfully delivered in 24 h. As expected, GEN contained in the baseplate was delivered through the created pores after the dissolution of the needle tips. On the other hand, the results obtained in the *in vivo* experiment confirmed that the use of dissolving MN arrays enabled transdermal delivery of GEN. It is important to highlight that once the GEN Cmax was reached, regardless of the dose, GEN levels in plasma were shown to be sustained over time. This may be indicative of drug still being released from the MN patch while concurrently being cleared from the body of the animals. It is important to notice that GEN is contained in the needle tips and in the baseplate of the MN array. And after the dissolution of the needle tips, GEN continued to be administered from the baseplate as the patches were covered by an occlusive dressing. Consequently, MNs provide a sustained release and that explains why the AUC obtained for MN administration are higher than the AUC obtained for the IM injection. Besides, this explains why the T_max_ is higher for the lower GEN dose administered using MNs. In this case, GEN delivered from the baseplate is required to reach the higher maximum drug levels. The patches with higher GEN doses contained enough drug in the needle tips to achieve the maximum drug levels within shorter periods of time.

As described before, this study was planned as a proof of concept and after evaluating the *in vivo* results it is obvious that the system should be optimized for further applications. In order to achieve a rapid delivery and prevent the sustained delivery the MN arrays can be manufactured containing the drug only in the needle tips. In this way, the drug can be administered in a quicker way preventing sustained release of GEN from the baseplate. Thus, drug clearance in animals will require further investigation. Regardless of the number of MN arrays used, it was observed that MNs were completely dissolved when arrays were removed from the back of the animal. Transient mild erythema was observed after removing the patches and, in line with previous publications, the erythema disappears quickly after MN removal [53], [54]. It is worth noting that concentrations of the HA, a FDA-approved biodegradable and biocompatible polymer, and low MW PVP, a biocompatible FDA-approved pharmaceutical excipient, were low in the formulation investigated. However, elimination of both polymers from the body should be investigated.

The therapeutic plasma levels of GEN in humans ranges between 4 and 10 μg/mL [8], [55]. GEN exhibits a concentration-dependent bactericidal effect, in which a linear relationship exists among higher peaks, the minimum inhibitory concentration ratio and improved clinical response [6]. With once-daily administration, higher peak concentrations may be observed. Peak and trough GEN serum concentrations should be therapeutically effective, while being nontoxic. Prolonged peak serum concentrations above 10–12 μg/mL and trough serum concentrations above 2 μg/mL should be avoided, as these levels may be potentially toxic [8]. Predicting pharmacokinetics from animals to humans require further investigation. However, one can prudently extrapolate the information obtained from the animals and approximate the patch size necessary to use in neonates. A neonate has an average body weight of 3 kg, which is approximately 15 times greater than the average body weight of rats used in this experiment (0.209 kg). For the medium dose treatment group, which applied two MN arrays of size 0.45 cm^2^, the < 1 cm^2^ MN patch was capable of delivering GEN achieving a mean plasma concentration of 5.34 μg/mL at 2 h upon application. Consequently, a patch size of approximately 15 cm^2^ could potentially deliver therapeutic doses of GEN and should be removed after 3 h to avoid possible GEN side effects. For the high dose treatment, the GEN Cmax was observed at 1 h post-application, reaching therapeutic levels. Thus, the high dose treatment group yielded a more rapid delivery of GEN, and the data showed less variability than the data obtained from the medium dose treatment group when the GEN Cmax was observed. Therefore, a patch size not larger than 30 cm^2^ could potentially deliver therapeutic doses of GEN in newborns and should be removed after 1–2 h in order to avoid oto- and nephrotoxicity. Nevertheless, Tanira et al. have reported shorter half-lives and larger clearances of GEN in young rats (2–3 months old) compared to older rats (22–24 months old) [56]. This is unsurprising due to small mammals such as rats tend to metabolise some drugs more rapidly than larger mammals [57], [58]. In addition, GEN pharmacokinetics is affected by renal function and neonates present reduced glomerular filtration rate. Therefore, it is expected that neonates have lower renal clearance of GEN compared to older infants and adults [59], [60], [61], [62]. Considering this, the estimated patch sizes could probably be reduced significantly. A transdermal patch of such size could potentially be applied to the thigh or to the upper back of the neonate, as both sites provide a relatively flat surface. The manual application of larger than 1 cm^2^ MN patches is feasible as shown recently by Ripolin et al. [53]. The acceptability and feasibility of delivery of GEN MN arrays at these sites should be assessed. A range of patch sizes would be required according to different weight categories. MN arrays allow the delivery of GEN without the need of injections and could enable expanded access to treatment outside of inpatient facilities. Additionally, this approach reduces dangerous sharps waste as dissolving MN arrays are self-disabling and cannot be reinserted into another patient after being used [11]. Future work will focus on optimisation of the two-step method to cast only GEN formulation in the tips of the MN arrays. A transdermal MN patch design with targeted GEN loading may reduce the risk of prolonged administration of GEN and toxicity if the patch is inadvertently worn too long.

## 5 Conclusion

The work presented here reports the successful formulation and mechanical characterisation of dissolving MN arrays containing the antibiotic, GEN. Furthermore, in *in vivo* experimentation, therapeutically relevant doses of the antibiotic were delivered to rats, highlighting the potential for exploitation of this delivery route for GEN administration. This promising technology could simplify administration of GEN in developing countries, thus expanding access to lifesaving outpatient antibiotic treatment for infants with severe infection during the neonatal period. This is an exploratory study and before this technology can be applied to patients several studies need to be conducted such as a formulation stability evaluation, a comprehensive pharmacokinetic study and patient usability/acceptability study. Finally, evaluating the potential pathway to commercialisation of this technology, and the cost of GEN-loaded MN arrays when manufactured at scale, will also be important factors in the potential health impact of this delivery approach in resource-limited settings.

The following is the supplementary data related to this article.

Supplementary Fig S1 Representative OCT images showing *in vitro* dissolution kinetics of a 19 × 19 MN array prepared using F1 and inserted manually in dermatomed neonatal porcine skin. The white scale bar at top right represents a length of 1 mm. Supplementary Fig S1
