# Supplementary material for: Transdermal delivery of gentamicin using dissolving microneedle arrays for potential treatment of neonatal sepsis
Source: J Control Release. 2017 Nov 10;265:30–40. doi: 10.1016/j.jconrel.2017.07.032 (PMC5736097; doi:10.1016/j.jconrel.2017.07.032)
Supplement: Supplementary Fig S1 — Representative OCT images showing in vitro dissolution kinetics of a 19 × 19 MN array prepared using F1 and inserted manually in dermatomed neonatal porcine skin. The white scale bar at top right represents a length of 1 mm. [file mmc1.docx]

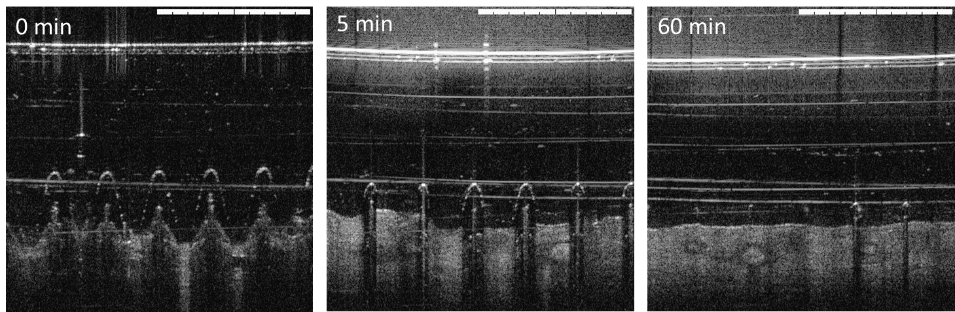


**Figure S1.** Representative OCT images showing *in vitro* dissolution kinetics of a 19 x 19 MN array prepared using F1 and inserted manually in dermatomed neonatal porcine skin. The white scale bar at top right represents a length of 1 mm.
